# A Rooted Net of Life

**DOI:** 10.1186/1745-6150-6-45

**Published:** 2011-09-21

**Authors:** David Williams, Gregory P Fournier, Pascal Lapierre, Kristen S Swithers, Anna G Green, Cheryl P Andam, J Peter Gogarten

**Affiliations:** 1 Department of Molecular and Cell Biology, University of Connecticut, Storrs, CT 06269-3125, USA; 2 Department of Biological Engineering, Massachusetts Institute of Technology, Cambridge, MA 02139, USA; 3 Biotechnology-Bioservices Center, University of Connecticut, Storrs, CT 06269-3149, USA

## Abstract

**Abstract:**

Phylogenetic reconstruction using DNA and protein sequences has allowed the reconstruction of evolutionary histories encompassing all life. We present and discuss a means to incorporate much of this rich narrative into a single model that acknowledges the discrete evolutionary units that constitute the organism. Briefly, this Rooted Net of Life genome phylogeny is constructed around an initial, well resolved and rooted tree scaffold inferred from a supermatrix of combined ribosomal genes. Extant sampled ribosomes form the leaves of the tree scaffold. These leaves, but not necessarily the deeper parts of the scaffold, can be considered to represent a genome or pan-genome, and to be associated with members of other gene families within that sequenced (pan)genome. Unrooted phylogenies of gene families containing four or more members are reconstructed and superimposed over the scaffold. Initially, reticulations are formed where incongruities between topologies exist. Given sufficient evidence, edges may then be differentiated as those representing vertical lines of inheritance within lineages and those representing horizontal genetic transfers or endosymbioses between lineages.

**Reviewers:**

W. Ford Doolittle, Eric Bapteste and Robert Beiko.

## Open peer review

Reviewed by W. Ford Doolittle, Eric Bapteste and Robert Beiko. For the full reviews, see the Reviewers' Comments section.

## Background

The use of DNA and protein sequence residues as character states for phylogenetic reconstruction was a profound breakthrough in biology [1]. It has facilitated advances in population genetics and reconstructions of evolutionary histories encompassing all life with most of the molecular diversity found among microorganisms [2]. While progress in theoretical aspects of reconstruction has allowed more confident and detailed inferences, it has also revealed the necessity for caution, as these inferences can be misleading if methodologies are not applied with care. At the same time, exponentially growing sequence databases including complete genome sequences [3] have allowed a more complete picture of biological lineages over time to be reconstructed, revealing new aspects of the evolutionary process.

Substantial incongruities in gene histories and uneven taxonomic distributions of gene families within groups of organisms have challenged a tree-like bifurcating process as an adequate model to describe organismal evolution [4-6]. In addition, evidence is abundant that the evolutionary history of Eukarya includes numerous primary, secondary and tertiary endosymbiotic events often providing important traits such as photosynthesis [7]. These inferences have caused a shift in the consensus among evolutionary biologists towards a view that the horizontal transfer of genetic material relative to vertical inheritance is a major source of evolutionary innovation [5,8,9]. With a growing recognition for the need to represent more than just the lines of vertical inheritance, various alternative models have been suggested. These vary in detail but broadly describe a reticulated network representation of organismal relationships [4,6,10-12].

## The Rooted Net of Life

In this manuscript we present a model, the Rooted Net of Life, in which the evolutionary relationships of organisms are more fully described than in existing Tree of Life concepts [13,14]. Importantly, we address the observation that organisms consist of many discrete evolutionary units: open reading frames, operons, plasmids, chromosomes and in some cases plastids and other organelles, each with discrete and possibly different evolutionary histories. These multiple histories are combined and plotted as a single reticulated network phylogenetic representation in which misleading artifacts of reconstruction and loss of information due to the averaging of phylogenetic signals are minimized. In some instances it may be possible to assign some edges as representative of ancestral vertical descent by genetic inheritance and other edges as reticulations due to horizontal genetic transfers. In other instances, this decision is less certain, for example, did the ancestor of the Thermotogales acquire the ribosome from a relative of the Aquificales, or did the Thermotogales acquire most of their genes from the clostridia? (See "Highways of Gene Sharing" below for details.)

Despite the distinct evolutionary histories among the genes in an organism, when they are found together in an extant genome, they are assigned to the same terminal node and edge that remains intact until their histories differ. This organism-genome definition includes histories of endosymbioses, which evolved to a point of bidirectional dependence *e.g.*, mitochondria and plastids with the "host" cell [7], but excludes parasitisms and mutualisms in which partners are facultative or interchangeable *e.g.*, the gut microflora of animals [15]. Ribosomal RNA and protein sequences are combined into a supermatrix and used to infer a well-resolved phylogenetic tree scaffold which we anticipate to mostly, but not necessarily, approximate the vertical descent of a coherent biological entity (but see the "Endosymbioses" section below). One terminal node may represent a group of sequenced genomes sharing very similar ribosomal sequences. All other genetic sequences including plasmids and chromosomes are assigned to tips by membership within these ribosome-defined pan-genomes and are further grouped into homologous gene families across other tips. Reconstructed phylogenetic trees of each are superimposed on top of the scaffold, forming reticulations where necessary.

## The Ribosomal Tree Scaffold

The complex relationship between individual genetic components and the evolutionary history of organisms must be well understood in order for a biologically meaningful, comprehensive history of life to be assembled from molecular data. Since species are propagated by the reproduction of individuals within a population, and generated by the divergence of populations over time, cytologically speaking, a single vertical tree of descent exists, at least for prokaryotes that procreate through division of the parent cell. However, in principle, this "tree of cellular divisions" [16] (ToCD) can only be indirectly inferred from molecular data, as opposed to gene trees, which are, in practice, explicitly described by molecular phylogenies. As such, the ToCD is only knowable insofar that a vertical signal is preserved; if all gene histories were dominated by random horizontal transfer, there would be no connection between cellular and genetic history. Additionally, the ToCD concept fails when a new cell is created through the fusion of two cells. If this fusion is part of the sexual life cycle, the principle of the ToCD is violated, but the deviations may be inconsequential if phylogeny is considered at a larger scale. However, instances of symbioses that lead to lineage and/or cell fusions between divergent partners (as in the serial endosymbiosis theory for eukaryogenesis, if mitochondria and plastids are no longer considered individual cells) lead to reticulations in the ToCD. Therefore, when all life is included, the ToCD does not represent a strictly bifurcating process.

Bridging the gap between gene and species trees has traditionally been approached via two methods: (1) supermatrix methods, which seek to infer a species tree by the concatenation of a large number of genes, integrating across many sites within aligned sequences to arrive at a well-supported, comprehensive tree [17]; and (2) supertree methods, which integrate across phylogenies calculated for many individual genes [18]. Both methods attempt to arrive at a consensus phylogeny to approximate the species tree by overcoming the insufficient and occasionally conflicting phylogenetic information that each molecular unit (typically genes) can provide. However, if applied indiscriminately, biased horizontal gene transfer can invalidate these methodologies, as multiple strong, distinct phylogenetic patterns can exist within a dataset [10,19]. In this case, it is possible that the resulting phylogeny will not only be incorrect, but even contain bipartitions not supported by any subset of the data due to fallacious averaging between signals [20]. While these approaches acknowledge that a comprehensive history of life must take into account many individual gene histories, it is clear that, at best, this is insufficient for capturing the true complexity of the evolution of life.

In supermatrix approaches, to avoid averaging over phylogenies with conflicting phylogenetic signal, gene families with conflicting gene phylogenies are usually removed. This results in genome or species phylogenies that only represent a small fraction of the genetic information within each organism, the so-called "tree of one percent" [13,21]. While such empirical approaches naturally result in a dataset dominated by the ribosomal machinery, they are philosophically unsatisfying not only in that they disregard all other gene histories (many, if not most, of which will be congruent across most of the tree, with the possible exception of closely related groups where transfers are far more frequent), but also because they are not definitive; revisiting gene phylogenies and definitions of sequence similarity with more advanced techniques could always add or remove genes from the dataset, affecting the inferred conclusions. The history of accounting for horizontal gene transfer (HGT) within phylogenies shows a normalizing progression from the filtering of genomic "noise", to the cataloging of HGT events as unique exceptions, to the acknowledgement of HGT as a major force in evolution [5,9,22]. Acceptance of the relevance of HGT for reconstructing life's history also follows this progression, and any serious attempt to capture a universal evolutionary schema must include reticulations, not merely as a decoration, but as intrinsic and essential to the understanding of the whole.

However, it is clear that regardless of its primacy (or lack thereof), a reference tree representing a robust, consistent evolutionary signal is an essential initial scaffold for any such holistic effort. Such a reference tree should be not only highly resolved and robust against artifacts, but reflect a biological reality consistent with its central organizing role, as opposed to an empirically determined collection of genes which are solely defined by their universal presence. A ribosomal tree, derived from the concatenated sequences of both ribosomal RNAs and proteins, is well-suited to this purpose [4,23,24]. The high level of sequence conservation within the ribosome, combined with infrequent horizontal transfer of its constituent molecular elements between distantly related groups, makes this an ideal candidate for providing a scaffold reference phylogeny [22,25].

To verify the congruence of the evolutionary signal within the ribosome, highly supported bifurcations between all sets of ribosomal gene trees were compared, identifying cases where specific topologies were consistently in conflict with others. In such cases, the particular sequences for those species in the conflicted area of the tree would not be included in the concatenation, in order to avoid fallacious signal averaging within the dataset. The vast majority of comparisons showed no highly supported conflicts, while 23 intra-order conflicts were identified within 10 groups across three domains. As these groups tend to be highly similar to one another at the ribosomal sequence level, and do not challenge the relationships between larger phylogenetic categories that are of the most evolutionary interest in a ToL/rooted Net of Life (RNoL), these were preserved within the dataset. Additionally, three inter-order conflicts were detected, with *Methanosaeta thermophila *L29 showing strong support for grouping with Methanomicrobiales, and *Staphylococcus aureus *S19 and L5 showing strong support for grouping with Lactobacilliales. No inter-domain conflicts were detected. It is important to note that this methodology does not specifically detect horizontal transfers; rather, it simply identifies well-supported conflicts that would violate the assumptions necessary for a concatenated ribosomal dataset. As many ribosomal protein sequences are very short, there is limited phylogenetic information per protein, and the resulting tree topologies reflect this in their lack of resolution. Therefore, a stringent criterion is required for the identification of clear conflicts, as poorly supported conflicts within these trees may merely reflect a very weak power of detection for actual events.

The use of the ribosome in providing a scaffold for a Net of Life reconstruction is also fitting in that a recent study has also used universal ribosomal proteins for an empirical rooting of their respective universal tree [19]. In this study, ancestral reconstruction of ribosomal protein sequences identified a unique compositional signature along the branch on the bacterial side of the tripartition between the three domains. Compared with simulations and other parts of the tree, this branch showed a significant under-representation of amino acids presumed to be more recent additions to the genetic code (Tyr, Trp, Phe, Cys), and a significant over-representation of those presumed to be the most ancient (Gly, Ala). As the current state of the genetic code is a character shared between all domains, this signal should be preferentially detected on the branch closest to its formative state, that is, the branch that contains the root.

While, strictly speaking, this only explicitly roots the "ribosomal tree of life" [19], it is a reasonable starting point for rooting the reticulate phylogeny, as it serves to polarize the proposed scaffold, allowing the full complexity of reticulations in a comprehensive evolutionary history to also be rooted with respect to one another. The majority of molecular phylogenies rooted using ancient gene duplications placed the root in the same location (see review in [26]); and the deep split between Bacteria and Archaea is also recovered from genome-wide analyses using midpoint rooting of splits trees, and averaging over phylogenies of nearly universal protein families [27-29]. Interestingly, reconciliations of gene trees to the reference scaffold tree can also provide further support for the correct rooting, as alternative placements of the root should consistently force less parsimonious reconciliations, if incorrect. It may even be seen that a distinct subset of reconciliations for related genes are more parsimonious with an alternative rooting (*e.g.*, on the archaeal or eukaryotic branch), supporting HGT events occurring between the stem groups of each domain, which would be extremely difficult to infer otherwise.

## Examples of reticulations

There are many organismal lineages that have been involved in horizontal genetic transfers, some at frequencies sufficient to be considered highways of gene sharing [10,24], thus leading to many different gene histories in the chromosome(s) of one organism [8]. When these organismal histories are considered internally consistent and tree-like, conventional phylogenetic reconstruction methods that combine sequence data often reflect an average between distinct signals. This is especially a problem in those cases where highways of gene sharing between divergent organisms dominate the phylogenetic information retained in the analyzed genomes. Multiple endosymbioses have occurred in many lineages, therefore organismal histories are better represented by a Rooted Net of Life able to reflect both vertical descent and horizontal genetic transfers. Here we outline examples that demonstrate a bifurcating tree-like phylogeny as an inadequate depiction of the history of life.

### Horizontal genetic transfer

There are numerous important gene sharing events, some between members of different Domains of life, that are lost when only a singular tree of life is considered. These include inventions of new metabolic pathways, such as a single transfer event in which genes encoding acetate kinase and phosphoacetyltransferase were transferred to the *Methanosarcina *from cellulolytic clostridia allowing the use of acetate as a substrate for methanogenesis (acetoclastic methanogenesis) [30]. There are also many examples of gene transfers from bacterial to single celled eukaryotes. The Fungi acquired many genes involved in various metabolic processes from both the Proteobacteria and Actinobacteria [31-36]. The protozoan *Blastocystis*, found in various gut environments, has acquired genes involved in energy metabolism, adhesion and osmotrophy from bacteria. These transfers have allowed for successful adaptation of *Blastocystis *spp. to digestive environments [37]. Genes involved in organic carbon and nitrogen utilization, the urea cycle, cell wall silification and DNA replication, repair and recombination have all been transferred from bacteria to the diatoms [38]. Bdelloid rotifers, metozoan freshwater invertebrates, have acquired genes for a xylosidase, cell wall peptidoglycan synthesis and various reductases and dehydrogenases from bacteria [39]. A pivotal gene transfer from the bacteria to the Cnidarians allowed for the development of the stinging cells that this lineage uses to capture prey [40]. The gene encodes a polyanionic polymer (PGA), which, when present in large quantities in the stinging cells (nematocysts), causes an explosive, stinging discharge to be released upon contact [41]. Examples of gene transfers from bacteria to multicellular eukaryotes include ancestral bacterivorous nematodes acquiring cell wall degradation genes from a bacterial lineage [42-44]. These genes are required for the initial step in parasitizing plants, enabling the free living nematode to "transition" into a parasite [45]. Other examples include *Wolbachia *endosymbiont sequences in the X chromosome of the host adzuki bean beetle [46] and in the *Aedes aegypti *genome [47].

### Highways of gene sharing

There is evidence that Thermotogales have a significant portion of their genomes transferred from the Firmicutes and Archaea, about 48% and 11%, respectively [48]. Averaging across the whole genome with supertree or supermatrix methods places the Thermotogales with the Firmicutes [48,49] and neither highways of gene sharing, nor the history of the ribosome emerges from the averaged signal. A similar case is seen for the Aquificales, which according to averaging methods are placed with the Epsilonproteobacteria, apparently due to an overwhelming number of HGTs from that group [50]. 16S rRNA gene trees and concatenated ribosomal gene trees place both the Thermotogales and the Aquificales, as deeply branching bacterial lineages [48,50]. Other examples include the Thermoplasmatales, an acidophilic euryarchaeal order, with about 58% of their genome inferred to have been transferred from the phylogenetically distant crenarchaeal Sulfolobales [51-53]; and *Methanosarcina mazei*, with about 33% of its genome identified as transferred from bacteria [54]. Such examples continue to emerge, and more are likely to be discovered as the number of sequenced genomes increases.

### Endosymbioses

We consider an organism to be a group of distinct evolutionary units currently engaged in an obligate mutualism. Thus we include the bacterium *Thermotoga petrophila *with its set of ancestrally archaeal genes as a single organism, assigned to a single terminal node on the Rooted Net of Life. Likewise, we would consider an animal with its numerous mitochondria-containing cells or a plant with its many mitochondria-and chloroplast-containing cells as respectively assignable to terminal nodes.

The events that led to these relationships can be considered large-scale horizontal genetic transfers in which an entire chromosome, along with a cell membrane, is engulfed *via *endosymbiosis. Subsequent evolution leads to an obligate mutualism [55] with gene transfer from the endosymbiont chromosome to the host nuclear chromosomes [56]. The primary endosymbiosis leading to plastids refers to an original uptake and retention of an ancestral cyanobacterium by an ancestral eukaryote [57]. Extant organisms retaining this ancestral condition are the Glaucophytes, Red Algae and Green Algae. Other lineages underwent secondary and even tertiary endosymbioses [7] providing not only prominent morphological features but also defining metabolic pathways (*e.g.*, photosynthesis). In tracing the genealogies of these discrete evolutionary units, numerous reticulations within the ribosomal tree scaffold itself are necessary, and these reticulations are congruent with the lineages of other genes present on the endosymbiont chromosome. These examples illustrate the reticulate complexities within all Domains of Life, and show that the assumption of a single, bifurcating organismal tree is problematic not only within specific groups of prokaryotes. However, to say the history of life is better represented by a Rooted Net of Life is not to say there is no structure or form to it; rather, that the structure and story is too complex for a single tree-like narrative to contain [58].

## Reconstructing the Rooted Net of Life

Phylogenetic reconstruction suffers less stochastic error when more data are available for most branch-length scenarios [59]. In reconstructing the Rooted Net of Life model proposed here, whole-genome data sets are required to provide both the tree-like ribosome scaffold and the potential reticulations from other gene trees. One extreme approach for mitigating stochastic error would be multiple whole-genome alignments, but this would not be realistic (or even possible given the incomplete homology of gene families across extant life) because the discrete evolutionary histories within organisms would not described. Where regions of a genome are likely to have had the same histories, combining sequences to improve resolution is a useful approach and is discussed in detail below. It is important to note that even well resolved phylogenies may be deceptive, with reconstruction artifacts masking complex evolutionary events if the reconstruction model was inadequate to describe the evolutionary process [60]. This is especially likely when incorporating diverse homologous sequences as is necessary in a Net of Life reconstruction.

### Mitigation of stochastic error: combining sequences for improved resolution

**Figure 1:**
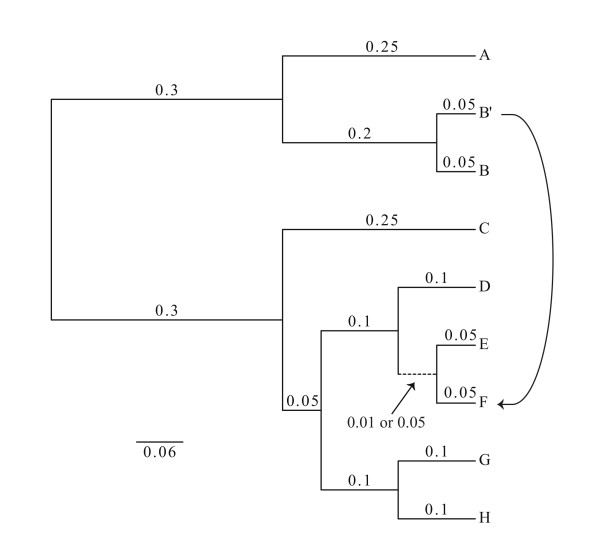
**Phylogenetic tree used to simulate genome evolution including a directed highway of gene sharing**. Two different trees were tested, one having a slightly longer internal branch of 0.05 substitutions per site compared to the other tree with only 0.01 substitutions per site. Genome B' was used as a donor for genes transferred into the lineage leading to genome F. Genome B' was not included in the phylogenetic reconstruction and genes from genome B' were used as replacements for their orthologs in genome F. The simulations were repeated with increasing amount of transfers from genome B' to F. The genome sequences were generated using Evolver from the PAML package [113]. Each simulated genomes contained a total of 100 genes, each 300 amino acids long.

**Figure 2:**
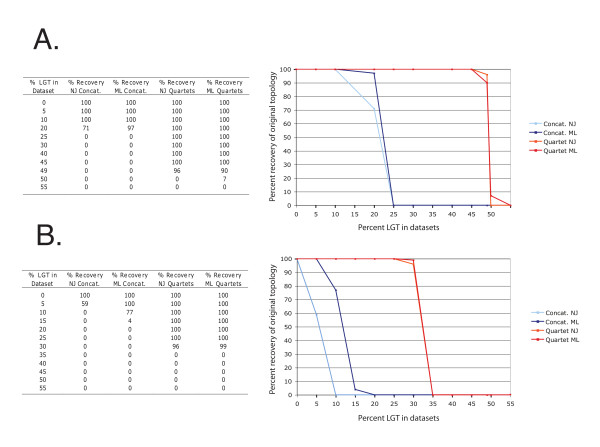
**Comparison of supermatrix and supertree approaches for recovering the correct tree following horizontal genetic transfer**. Horizontal genetic transfer was simulated between lineage B' and F (Figure 1) with an internal branch of 0.05 (A) or 0.01 substitutions per site (B). The frequency with which the correct tree is recovered from supermatrix and supertree approaches from data that include increasing amounts of genes transferred along a single highway of gene sharing was tested. Each simulated genome contained a total of 100 genes, each 300 amino acids long. Genes were concatenated into a single sequence from each simulated genome for the supermatrix tree calculation or alternatively, gene trees were calculated individually from each gene for the supertree approach. The sequences were not realigned to avoid any additional artifact potentially introduced from alignment algorithms. Neighbor-joining trees were calculated with Kimura correction in ClustalW version 2.0.12 [114]. Maximum likelihood trees were calculated with PhyML V.3.0 [115] with Pinvar, JTT model and estimated gamma distribution under 4 categories. The embedded quartet trees [116] as well as the resulting plurality trees (supertree) were calculated from the individual gene family trees using Quartet Suite v.1.0 [117]. The simulations were repeated 100 times to measure the reproducibility of the different tree reconstruction methods in recovering the original tree topology.

To solve difficult phylogenies, it is sometimes advantageous to use information from many genes in order to extract phylogenetic signals which otherwise may be too dilute if taken from individual genes. As previously mentioned, two widely used methods consist of concatenation of multiple genes (supermatrix) [17] and construction of consensus phylogenies using several trees calculated from individual genes (supertrees) [18]. It is believed that these phylogenomic methods are capable of capturing a plurality consensus of a dataset while minimizing the presence of artifacts in the data such as presence of gene transfers or low phylogenetic signals. However, if too many conflicts are present in the datasets or the phylogenetic signal is too weak, the resulting consensus tree may not be informative, as it may not accurately reflect the history of any of its constituent datasets [61]. This can be illustrated using simple genome simulations involving a single highway of gene sharing between two unrelated lineages (Figure 1) where supertrees based on embedded quartet decomposition outperformed gene concatenations (Figure 2). When genes were transferred to a lineage whose neighboring branch was separated by 0.05 substitutions per site (Figure 2A), the supermatrix approach (concatenation of genes) was able to recover the correct tree topology only when less than 25% of the genes underwent homologous replacement. In contrast, embedded quartet decomposition followed by supertree reconstruction recovered the correct topology, even when 45% of the genes underwent HGT replacement (Figure 2A). At more than 50% HGT, genome F was recovered as the sister group to B, reflecting a situation where the signal due to ancestry is overwhelmed by a highway of gene sharing. When the recipient lineage is positioned closer to its sister group, the supermatrix approach was even more susceptible to HGT (Figure 2B). The presence of 10 to 15% of misleading signal in the concatenated dataset was sufficient to induce the recovery of the wrong topology in the majority of cases. In the same situation, the quartet based supertree approach failed in the presence of 35% or more of conflicting signals. In contrast, when no gene transfers were simulated and the amount of phylogenetic signal varied only between datasets, supermatrix approaches fared better in extracting the correct phylogenetic signal compared to supertrees (data not shown).

These results indicate that when using sets of genes that are known to be less frequently transferred, as may be case for ribosomal proteins, a supermatrix approach is preferable, whereas for datasets where cryptic highways of gene sharing may connect divergent organisms, supertree approaches such as quartet decomposition may be more accurate. An additional source of error caused by the stochastic way in which lineages sort during speciation can result in anomalous gene trees in phylogenetic inference [59]. This can arise during periods of rapid diversification where short edges are present in gene trees and is not mitigated by combining more genes into a single analysis.

### Accounting for heterogeneous evolutionary processes

The reconstruction of phylogenetic trees from biological sequences relies on estimation of the evolutionary distance between the sequences of interest. This estimate is obtained from evolutionary models that describe the probability of different nucleotide or amino acid substitutions [62]. Traditional evolutionary models are based on a set of simplifying assumptions, and when these assumptions are violated by the dataset examined, incorrect trees may be recovered [62,63]. In phylogenetic reconstruction on a RNoL scale, where a large degree of sequence diversity is included, these simplifying assumptions run an even greater risk of violating observed biological realities not explicitly described within the reconstruction model. Some of these challenges to evolutionary models are described below, along with the work being done to overcome them.

Extant lineages can substantially differ in base and amino acid composition, a phenomenon known as compositional heterogeneity [62,64]. In many cases, this is driven by physiological adaptation to environments with distinct demands on protein physiochemistry (*e.g.*, thermophily, halophily). Changes in nucleotide composition of the genome (*e.g.*, high or low G+C content) can also occur within specific lineages, indirectly affecting amino acid composition. Models that assume compositional homogeneity (constant sequence composition throughout the tree) tend to group lineages with similar compositions together, regardless their actual evolutionary history, and produce high bootstrap values for these incorrect topologies [62]. A solution to the problem of describing compositionally heterogeneous datasets is the implementation of models that allow for different equilibrium frequencies (parameters to describe sequence composition) on different parts of the tree [62,64].

Another challenge for evolutionary models is heterotachy, the variability in evolutionary rate at a site on different branches of the tree [63]. Heterotachy can cause evolutionary models to group taxa on long branches together, affecting both maximum parsimony and maximum likelihood methods [65], and producing incorrect trees with high bootstrap support [63]. The deleterious effect of heterotachy on phylogenetic reconstruction can be mitigated by the use of probabilistic models with sufficient parameters to correctly describe this phenomenon [63,65].

Most current evolutionary models are also ignorant of secondary and tertiary structure - that is, they assume that substitutions at one site are completely independent of substitutions at another, an assumption that is violated by the sequence evolution of protein and ribozyme coding genes (including ribosomal RNA). Models of nucleotide substitution that weigh the rate of nonsynonymous nucleotide substitutions by their effect on protein tertiary structure [66], or that estimate the variation in nonsynonymous substitution rate in a sequence [67], are being developed. These models show promise, especially for the detection of positive selection, but remain computationally expensive and are outperformed at phylogenetic reconstruction by site-independent models [68]. Accounting for structural information is also known to improve RNA alignments, especially in divergent sequences [69], and models that account for secondary structure when performing phylogenetic reconstruction are under development. These models improve phylogenetic trees in some situations [70], but produce incorrect results in some others [69]. Nevertheless, they show promise and deserve further investigation.

Improvements to evolutionary models are constantly being made, and lead to improved ability to distinguish phylogenetic information from noise. These new models increase the number of parameters used to describe the data, and this strategy is merited in many cases. However, it is important to recognize that adding unimportant parameters decreases the power to draw conclusions [64], and that not all datasets will be best described by the same model. Including more parameters does not necessarily improve reconstruction - for example, evolutionary models that use different parameters for each branch of the tree are often outperformed by models that allow for only two different sets of parameters, one for each major clade on a tree [64,71]. As evolutionary models are being developed and improved, it is important that methods for selecting the best model for a dataset also be explored [71], as has been done in some cases [64], and developed for use by wider audiences.

Other artifacts can also be present within reconstructions, independent of rate and composition model parameters. Longer branches will tend to group together regardless of their true relationships [72], a phenomenon seen in the artifactual placement of microsporidia as a deep branching eukaryotic lineage [73,74]. Periods of rapid diversification causing shorter branches will leave reconstruction vulnerable to the node-density effect where branch lengths may be overestimated in areas of the tree with more nodes [75]. Although balanced taxon sampling may mitigate some of these artifacts, the course of evolution is not obliged to supply phylogenetic distributions that are easily reconstructed across the whole Net of Life [73], thus the development of improved algorithms is an important area of research.

### Acknowledging diversity within the Rooted Net of Life

Biological evolution has manifested itself in an impressive array of diversity. Life histories among organisms vary widely with corresponding differences in population dynamics and modes of diversification ("speciation"), perhaps most significantly between unicellular and multicellular lineages. These two groups differ greatly in their propensity for horizontal genetic transfer with implications for the interpretation of gene tree conflicts. For multicellular organisms with somatic cell lines, the probability of horizontally transferred genetic material being copied into the progeny of the host is much lower than for unicellular organisms. However, examples of the former do exist. As noted above, these are often transfers from a bacterial symbiont to the host genome. Interpretation of gene trees conflicting with the backbone reference tree should thus be informed by life histories and other prior biological knowledge of the lineages concerned: a conflicting topology among unicellular taxa is more likely to be due to HGT than is a conflict among multicellular taxa where an alternative hypothesis of differential gene loss or incomplete lineage sorting may be preferred.

When considering macroevolutionary relationships, conflicting topologies within closely related groups, which are more likely even for ribosomal genes, will not change the deeper relationships. Of 568 species of Bacteria and Archaea represented in the NCBI Complete Microbial Genomes database in late 2009 [76], 235 had diversity among multiple 16S rRNA copies [77]. In the majority of cases intragenomic sequence diversity is less than that conventionally defined for interspecies diversity [78]. Of the 2.5% of species with sequenced representatives that exceeded the interspecies limit [77]*Thermoanaerobacter tengcongensis *with 6.7% diversity and certain lineages of Halobacteriales including *Haloarcula carlsbadense *[79] and *Halomicrobium mukohataei *JCM 9738(T) [80] are of particular note. While resolution at deeper levels would be unaffected, there is sufficient divergence in this small minority potentially to cause resolution problems at the genus level. The use of a supermatrix including ribosomal proteins, which are single copy genes [77], would mitigate this. Thus the use of ribosomal sequences (protein and rRNA) as a scaffold of mostly vertical descent onto which a Rooted Net of Life can be inferred is not negated. However, the correlation between scaffold and vertical inheritance is not inviolate, or essential to the construction of such a Rooted Net: the transfer of an entire ribosome may be inferred by a topological incongruity between the initial scaffold and a large majority of the other gene phylogenies associated with that lineage.

### Reconciling gene histories

Various approaches for obtaining a single supertree from several gene trees within the same set of genomes (sometimes referred to as a "species tree" in the literature) have been proposed [81-83]. As emphasized above, such approaches are only appropriate for situations where HGT between divergent lineages is unlikely - either because of the nature of the lineages considered (multicellular) or the nature of the sequences used (*e.g.*, ribosomal). Rather than infer a new topology representing a "species" tree, related algorithms have been developed by Beiko and Hamilton [84] and Lawrence and Alm [85] using a predetermined reference topology with similarities to the model proposed here. In the latter, through a process called "reconciliation", gene tree topologies are chosen that both support the sequence data and minimize a cost function determined by gene loss, gain and transfer relative to a reference phylogeny. Reticulations representing HGT are therefore accommodated, although unlike the model proposed here, the initial topology exclusively and explicitly represents a history of vertical descent. For this reason, even if the initial reference topology is carefully chosen, a simple application of this approach has a limited capacity to reflect a comprehensive evolutionary history of life. However, these approaches can be accommodated within the RNoL model by removing assumptions equating the reference tree with vertical inheritance, and extending subsequent analyses to take more complex events into account, such as those previously described (*e.g.*, endosymbioses, lineage-specific trends of HGT *vs*. duplication). In these models as in the RNoL, there will be an inevitable "thinning" of edges towards the root because of genetic losses (genes, plasmids, organelles *etc*). Assigning these losses to HGT events or to lineages of vertical descent will not be possible in regions of lower phylogenetic resolution where there are ambiguities associated with HGT; but in principle this model provides a retrodictive representation of biological evolution

## Conclusion

As more genome sequence data have become available and are analyzed, evolutionary biologists and philosophers have begun to question the legitimacy of the Tree of Life concept. Various analytical approaches for dealing with the newly inferred and distinctly non tree-like nature of organismal lineages have been presented with differing underlying assumptions with respect to the nature of the evolutionary process [28,58,86-88]. We have described a Rooted Net of Life model of evolution, accommodating the numerous examples of reticulated histories, that is better able to describe the history of life than the pervasive Tree of Life concept while retaining retrodictive power. Retrodiction is lost in some alternative propositions that phenetically cluster extant organisms by patterns of diversity left by the evolutionary process. The macromolecular sequences of the ribosome, homologous in all cellular life, provide the information to reconstruct an initial scaffold of predominately, but not necessarily, vertical descent. This averages over many reticulations at lower taxonomic levels, and includes a few large-scale reticulations where the ribosomes in the eukaryotic organelles are mapped to the same tips as those of the nucleocytoplasmic components. All other genetic sequences can then be recruited to combine with this ribosome-based scaffold to more fully depict and better define both the vertical and horizontal components of the history of life.

## List of abbreviations

RNoL: rooted net of life; HGT: horizontal genetic transfer; ToCD: tree of cellular divisions; ToL: tree of life.

## Competing interests

The authors declare that they have no competing interests.

## Authors' contributions

JPG, GPF and DW conceived of the study. PL, GPF and CPA performed data analyses. JPG, DW, GPF, PL, KSS and AGG participated in its design and wrote the manuscript. All authors read and approved the final manuscript. GPF and DW contributed equally.

## Reviewers' comments

### Reviewer 1: W. Ford Doolittle, Dalhousie University

"Rooted Net of Life" might well be the right name for what I suspect is currently the most popular way of thinking about microbial phylogeny within the systematics and evolution community, and Williams *et al. *do a fine job of articulating this view as a model. Still, some critique seems called for.

First, one might object that there is a conflict with the other paper from the Gogarten lab included in this special thematic series of Biology Direct. If gene transfer can be so biased as to assume responsibility for certain aminoacyl tRNA synthetase tree topologies - which I take to be the import of the Andam and Gogarten submission - then why do we not also assume that to be the case for genes that do not so readily lend themselves to analysis as do those homeoallelic exemplars? And why do we assume that "phylogenetic bias" so often trumps other sorts of physiological, ecological or geographical biases? No doubt the Tree of Life, constructed by either supermatrix or supertree methods (which Willams *et al. *distinguish very nicely) tells us *something *about central tendencies in prokaryotic evolution, but it is only the "complexity hypothesis" that holds out some promise that the first of these methods might give us something like the Tree of Cell Divisions.

**Authors' response: ***To avoid confusion, we briefly want to summarize the interplay between HGT and our rooted Net of Life proposal. In light of the homeoallelic exemplars and other evidence for biased gene transfer *[89-91]*, we indeed need to reconcile our proposal to the possibility of phylogenetically biased transfers*.

Transfer of ribosomal components between close relatives: *Undoubtedly, highly conserved ribosomal components are frequently transferred between close relatives and following transfer are integrated into the recipient's genome. At least for ribosomal RNAs, it was shown convincingly that a gene acquired through transfer does recombine with the homolog already present in the recipient (see discussion in *[22,92]*and *[93]* for examples), thus turning the ribosomal RNA into a mosaic. However, most of these transfers are indeed between close relatives and only become detectable when many genomes of close relatives are analyzed. The proposed ribosomal scaffold averages over these transfers and subsequent recombination events. Consequently, the transfers between close relatives will only rarely affect the relative placement of families and higher taxonomic units; however, the scaffold may be an unreliable reference for within family and within-genera phylogenies*.

Transfer of ribosomal components between divergent organisms: *Screening individual ribosomal protein families for phylogenetic conflict, and assigning the sequences from the recipient and its descendants to different data partitions, will avoid averaging over transfers between less related organisms. However, individual ribosomal proteins contain little phylogenetic information, and thus this screen will be unreliable for within-family transfers. The ribosomal scaffold will tell us about the central tendency of the ribosome, after removing transfers between divergent organisms (such as described in *[94]*) from the averaging. This scaffold is not intended to tell us anything about the central tendency of the genome or of the organism. If for part of the phylogeny the central tendency of the genome agrees with the central tendency of the ribosome, then there is no indication for highways of gene sharing that are not biased by close relationship. If the two conflict, such as in case of the extreme thermophilic bacteria, we can conclude that genes were transferred with a bias determined by other factors such as the ecological niche. We cannot distinguish a priori the transfer of the ribosome from a highway of gene-sharing through which the majority of genes were transferred; however, increased taxon sampling may detect transfers spread out over time, as would be expected for a transfer bias caused by a shared ecological niche, and thereby allow us to discriminate this from a single event leading to the formation of a chimera between two partners*.

Trickle-down transfer *vs *shared ancestry: *We cannot exclude the possibility that an organism replaced its ribosome, either though acquisition of a superoperon in a single transfer, or through many transfer events that are biased not by close relationship (reflecting recent shared ancestry) but through other factors, such as a shared ecological niche. The ribosomal scaffold would place the recipient's ribosome close to the donating lineage. In case frequent transfer and recombination events occur within a group, individuals within this group in the ribosomal scaffold will appear more related to one another, and organisms not participating in the frequent within-group transfers may be left behind *[22]. *In either of these cases, the ribosomal scaffold does not represent the tree of cells but only the history of the ribosome. In many instances it will be possible to further elucidate the history of the genome, as is exemplified by the thermophilic bacteria *[48,50]*, and this might allow further inference regarding a likely tree of cells. However, the relationship between organisms is not sufficiently described by a single tree, and the RNoL provides a first step to elucidate the history. If the complexity hypothesis is true for the ribosomal components, the ribosomal scaffold may be similar to the tree of cell divisions. However, this is not a precondition to reconstruct the RNoL. Reconstructing the RNoL will identify those parts of life's history where a single tree of cell divisions provides an incomplete narrative*.

**Reviewer 1 continued: **Second, we might ask why the microbial systematics and evolution community still feels that we need some single way of describing the relationships of organisms and some singly historical "metanarrative" to undergird it. I'd guess our colleagues doing human linguistic, cultural and social history would see this as an unnecessarily simplistic and ultimately misleading aspiration (see for instance [95]). Is it just our need to defend Darwinism from its politically powerful opponents that causes us to cling to it?

**Authors' response: ***This is a fascinating question. In the context of this manuscript, we make the assumption that there is a single "true" sequence of events or organization of matter on the temporal and spatial biological scale (*i.e., *Life on Earth). The goal of reconstructing the resulting relationships between organisms is therefore to recover a single, historical description - but any such attempts are limited by the methods used and the data available (which at present do impose limitations on the confidence of historical events/relationships)*.

*Indeed, this proposed Rooted Net of Life is intended as a phylogeny of biological lineages that accounts for the horizontal exchange of genetic material and is composed from gene families found in sequenced genomes. It therefore has the same limitations as conventional phylogenetic comparative methods (it requires accurate alignments for homologous comparisons, three or more tips for a rooted reconstruction *etc*). We think a strength of this model is its direct depiction of evolutionary events allowing historical inferences rather than phenetic approaches (such as split-graphs representations or clustering genomes by genome content *etc*). which serve a different purpose in evolutionary biology*.

### Reviewer 2: Eric Bapteste, Université Pierre et Marie Curie

Peter Gogarten and his team play a major role in the debate on the Tree of Life (TOL). Therefore, their contribution to this special issue on how to go beyond the TOL is of unquestionable importance. They propose the reconstruction of a "rooted net of life" (rNOL) as a new reasonable goal for phylogenomics. In many respect, this notion seems sound: it is likely a research program that many phylogenomicists will be tempted to embrace. In particular, I entirely agree that organisms consist of many discrete evolutionary units, with multiple histories, a fact that is lost with the TOL, and therefore the TOL is not sufficient to capture true complexity of the evolution of life. It is also important to reckon that a universal evolutionary schema must include reticulations, not merely as decoration but as an intrinsic feature.

Two major comments however. First, the rNOL is not the only possible research path for evolutionists "beyond the TOL". Second, if embraced, important conceptual clarifications are still required to interpret the rNOL, because it cannot be done merely with the concepts of the TOL. A well understood rNOL is not just a TOL plus some fancy lateral edges, it is not quite "phylogenetic business almost as usual".

Major comments

1. The rNOL is not the TOL

This claim is crucial and should be made more significant, because it has practical and conceptual implications. The move from a TOL to a rNOL is more than just an extension of the TOL, through the addition of lateral branches to this tree. The rNOL research program really goes beyond the research program associated with the TOL. The former nodes and edges are not directly comparable to the nodes and edges represented in the TOL. Therefore the nodes and edges of the rNOL and of the TOL cannot really be interpreted alike. It would be misleading, therefore, and for the sake of convenience - a rhetorical trick - to describe the rNOL with the words and notions designed to analyze the TOL. Tree-thinking should not be directly imported en bloc into rNOL-thinking, as if not much was changing when the rNOL replaces the TOL to represent evolution. If the interest of evolutionists shifts from the TOL to the rNOL, some new concepts are needed to interpret the rNOL. This fundamental aspect of the transition from a TOL to a rNOL should be made much more explicit in this MS. I would like to suggest that the authors devote a short but entirely novel section to the issue of rNOL-thinking, that shows that going from the TOL to the rNOL requires significant (and not just minor) conceptual adjustments.

**Authors' response: ***We agree that adoption of the RNoL concept requires conceptual adjustments. Change is no longer gradual along a lineage, but often instantaneous due to HGT. Nodes no longer represent exclusively events of lineage divergence, but also the confluence of genetic information. Most microbiologists do recognize the importance of the processes that lead to reticulation, but only phylogeneticists have struggled to incorporate the diversity of biological processes into their reconstruction of evolutionary history. Given that processes of reticulated evolution are the focus of much research in microbiology, we do not think it necessary do devote additional space in the current manuscript to its discussion*.

**Reviewer 2 continued: **For example, the authors propose that each organism in a rNOL is represented by a single node and a single edge, unless the organism changes. For them a node is a meeting place for a possible genetic melting pot: the organism lies where various units join in a collective obligate mutualism. This notion of an organism is interesting, but is it the organismal notion associated with the TOL? I would say "no".

**Authors' response: ***By "terminal node" we mean to refer to the "tips" of the inferred gene and ribosome trees from which the network will be constructed. All sequences at these tips are taken from sequenced genomes (that is all chromosomes and plasmids sequenced from a sampled "organism") and so members of different gene families can be confidently associated with one another, at the tips, on that basis. This model is intended as a phylogeny as opposed to a more general clustering scheme based on evolutionary relationships. Internal nodes do, therefore, represent ancestral organisms inasmuch as the resolution of the data allows. Gene family members lost from an ancestral organism along a lineage cannot of course be represented *via *this comparative approach and thus internal edges and nodes can only be a partial representation of the genome complement of an ancestral organism. (Further inferences of what could be missing from such an inferred ancestral genome complement could perhaps be made though). It would be permissible to take a single ribosome as representative of a group of sequenced genomes (defined by ribosome gene sequence similarity) and include the pan-genome of those organisms in the same way*.

**Reviewer 2 continued: **Why does it matter? Because then the vertical backbone of the rNOL does not track organismal evolution. It tracks the evolution of the least mobile units of this collective obligate mutualism, or, if one wishes, it captures the "(less mobile) background organism".

**Authors' response: ***The reviewer makes an insightful observation here and below. However, something we perhaps failed to make clear in the original MS is that the ribosomal tree-shaped scaffold need not represent the line of vertical descent if the topologies of the other gene families suggest otherwise. In fact, where there is insufficient evidence to attribute any one set of internal edges to the line of vertical descent, we do not consider an agnostic attitude to be a problem. But we do anticipate that many of the edges will be less ambiguous and assignable as either representative of a horizontal genetic transfer or vertical genetic inheritance. The ribosomal scaffold serves only as an initial, well resolved rooted phylogeny with which other gene family phylogenies can be compared as a means of inferring a rooted net. The meaning of the term "reconciliation" as most often used in the literature (in the context of a "species tree" and several "gene trees") would be inappropriate here and so we agree the term "species tree" is best avoided. Another reason to object to the term "species" is the difficulty in applying the already troublesome idea of a macrobial species to the microbial diversity of which most of the RNoL consists*.

*However, we would suggest the term "organismal lineage" is not such a problem. As the reviewer suggests for the RNoL model, the identity of the organism will change along a set of "vertical" edges as nodes due to reticulations are crossed and genes are gained. This seems comparable to the accepted use of this term in a ToL model where the conceptual identity of an organism could change along an edge due to adaptation to a changing environment, or even more abruptly before and after a bifurcating speciation event*.

*We agree with the reviewer that these vertical edges, where identified, are likely to capture more of the "(less mobile) background organism"', because of the difficulty of mapping with any certainty to map the more mobile genetic elements to deeper edges. However, a vertical edge midway between the root and tip of the RNoL would in fact consist of many edges from the combined phylogenies of the gene families and ribosome. Tracing that vertical edge either towards or away from the root will cross nodes at which reticulations will leave or join it, so that all genomic components of an ancestral organism for which the phylogenetic comparative approach is suited will be represented, regardless of mobility. Notable omissions are discussed below*.

**Reviewer 2 continued: **However, with such a definition, the organism itself changes each time a new genetic unit (*i.e*. one or several genes, or a symbiont) comes in or goes out of the collective obligate mutualism. Therefore, in the rNOL every lateral connection in addition to the vertical splits gives rise to a new organism. New names are needed to describe these nodes, which do not exist on a tree. This in turn has an important consequence for another default notion of tree-thinking: the notion of (phylogenetic) species. Phylogeneticists cannot track species as easily on a rNOL as they were hoping to do on a TOL. What type of "chunk of the rNOL" corresponds to a species cannot probably be decided without considering what biological features the in-edges and out-edges provide or remove from the "background organism". In other words, not every edge (and not all sets of nodes/not every node) creates a new species. How is it decided what edge does and what edge does not define a new species? We need names to distinguish these edges. (And this is without mentioning the fact that sometimes "species" of interest lie in the very mesh of the lateral edges, precisely when gene exchanges are the defining criteria of an evolutionary unit one wishes to call a species rather than organisms with a conserved vertical core). As the rNOL would be a real opportunity to acknowledge the multiple processes at play in evolution, this clarifying goal is also part of this new research program. It likely requires creating suitable concepts, rather than importing "good old notions" that worked (to some extent) soley for the vertical process (*e.g. *the tree of cell division is not telling us where a species starts or ends, *etc*.). Advocates of the rNOL should therefore refrain from calling the vertical part of the rNOL the "species tree" or the "organismal tree": species/organisms may not be defined by vertical processes to start with. There are many reasons to give a more accurate name to that likely important vertical backbone, while not conflating it with a "species tree". I encourage the authors to rephrase their MS accordingly, where necessary, and to replace "species tree" or "organismal tree" or "TOL" by "vertical backbone" or by "tree of the least frequently transferred units" when that is what they mean. Discriminating a vertical backbone in the net of life matters, and calling it the TOL may limit the deeper meaning of the rNOL enterprise. (Interested readers can also refer to [96]).

**Authors' response: ***We agree with the reviewer and have updated the manuscript accordingly*.

**Reviewer 2 continued: **2. The rNOL presented here is a rNOC, but is the rNOC inclusive enough to describe evolution?

As it is described in the MS, the rNOL seems first concerned with the evolution of cells and that of cellular genomes. Where are the plasmids and the viruses in the rNOL? Is their evolution also modeled by it, and where? Or, unfortunately their evolution is not really represented, meaning that the rNOL has room only for cellular genomes and not all evolving elements with DNA genomes? It is unclear how the many plasmidic and viral genomes (some of which are without homologues to cellular genomes and to other plasmids and viruses), or even how ORFan genes, or all the sequences too divergent to be aligned and put in a tree, or the many environmental genes, could fit in a single rNOL. Where do they fit? The reference scaffold of the rNOL, based on ribosomal RNAs and proteins, seems largely to act as the reference phylogeny of ribocells [97].

**Authors' response: ***The limitations of the RNoL are the same as those of the comparative methods that are used to construct it. True ORFans (*i.e. *open reading frames that have no detectable homolog in any other genome) would not provide information on the topology but could be included in the model as tip metadata (quantified per genome). Comparison of the tips, each being all sequence data from a sampled organism or the pan-genome of a group of organisms with similar ribosome sequences, provides the internal topology*.

*Thus the contents of a plasmid can be treated in the same way as any other chromosomal gene: its position at the tips is defined by the other sequences sampled with it from an organism or group. We would expect to recognize reticulations leading from these gene trees closer to the tips than is typically found for chromosomal genes. Prophage sequences can be incorporated in the same way. Although tips are defined as organismal (pan)genomes, viral genomes are not in principle excluded and the reviewer makes a salient enquiry in this respect. The only limitation for inclusion is homology shared with enough for phylogeny reconstruction*.

**Reviewer 2 continued: **As such, the rNOL describes a larger part of the history of life than the TOC (tree of cells), yet it does not really describe the "full history of life". That's why it is important to acknowledge that going beyond the TOL could be achieved by using additional/alternative paths than the rNOL.

**Authors' response: ***In "The Rooted Net of Life" section we say "evolutionary relationships of organisms are more fully described than in existing Tree of Life concepts". This was the meaning intended in the conclusion but was miscommunicated in error and the manuscript has been revised. The reviewer is correct in pointing out limitations of the RNoL. While the RNoL provides an approach to reconstruct life's history, this reconstruction will often be ambiguous and incomplete. For example, at present no algorithm exists that would allow the reconstruction of ancient gene families that have left no extant descendants. While a complete reconstruction of life's phylogeny will likely be impossible, we believe that the RNoL will provide a more detailed and more accurate phylogeny than is possible under the ToL paradigm*.

**Reviewer 2 continued: **Other research paths are also possible beyond the TOL.

This is not a major criticism, simply an observation: the evolutionary literature about what evolutionists could do if the TOL were no longer their default option is a bit more heterogeneous than suggested in this MS. Some more literature could have been cited at places to put the rNOL solution retained by the authors in a larger scientific perspective. I can think of at least two very different options that were not discussed here, and I would like to encourage the authors to quote them somewhere in the slightly revised version of their MS:

a) Pattern pluralism [58] that questions whether we need to replace a unique representation by another unique representation. See also [98] that explicitly proposes to model different evolutionary outcomes with different evolutionary patterns (one tree, one rNOL, disconnected genome networks based on shared sequences, etc.). About these latter genome networks, see all the refs in [99], and the research program suggested in [100].

b) Analyses of phylogenetic forests [28,86-88]. Unrooted gene trees can be analyzed through various methods of tree-cutting, the most famous so far being the methods of quartet decomposition that can inform us about evolution without necessarily providing a grand rooted unified evolutionary scheme, or requiring the reduction to a single graph (tree-like or web-like).

I feel it is important to acknowledge that how to go beyond the TOL is itself debated.

**Authors' response: ***We added and discussed some of the suggested citations in the revised manuscript and we expanded the discussion of the RNoL concept. However, the goal of this manuscript was to propose an approach that allows reconstructing evolutionary history. There are many very useful approaches in comparative genomics that allow identification of genomic islands, molecular parasites, prophages and agents of gene transfer that are important in understanding microbial genetics and mechanisms of molecular evolution. However, these have only limited value for reconstructing the more ancient history of life. We already devoted a significant portion of the manuscript to discuss consensus tree approaches and their limitations; however, we do not think it will improve readability of the manuscript if we add a more detailed discussion of other approaches that use phylogenetic information retained in gene families to detect plurality and conflicting phylogenetic signals. We and others have co-authored manuscripts on this question in the past *[101,102]*, and the interested reader is invited to consult these and the manuscripts mentioned by the reviewer for further information on how to extract and use phylogenetic information from genome data*.

Reviewer 2 continued:

Minor comments

The authors indicate that "many, if not most of [the genes] will be congruent across most of the tree". I do not think we know that (most of the time this is not tested but assumed), and for the datasets that I tested I did not observe this kind of agreement. Rather most of the prokaryotic/viral/plasmidic genes are surprisingly incongruent. We will hopefully have some data published on that question in future works (Leigh *et al.*, in prep.), but the thousands upon thousands of microbial trees I had the opportunity to view are in my opinion more messy than suggested here. See also [103] for multiple phylogenetic histories in *E. coli *strains.

**Authors' response: ***As is now better described in the manuscript using more precise nomenclature, the objective of testing for ribosomal congruence was to determine to what extent the ribosomal proteins could be used as a rooted reference backbone tree upon which to map gene reticulations. To this end, we constructed phylogenies for ribosomal proteins (both universal core proteins and domain-specific proteins). Comparing highly supported bifurcations between all sets of trees, we identified cases where specific proteins were consistently in conflict with others. As such, the particular sequences for those species in the conflicted area of the tree would not be included in the concatenation, in order to avoid fallacious signal averaging within the dataset. The vast majority of comparisons showed no highly supported conflicts, while 23 intra-order conflicts were identified within 10 groups across three domains. As these groups tend to be highly similar to one another at the ribosomal sequence level, and do not challenge the relationships between larger phylogenetic categories that are of the most evolutionary interest in a ToL/RNoL, these were preserved. Additionally, three inter-order conflicts were detected, with *Methanosaeta thermophila *L29 showing strong support for grouping with Methanomicrobiales, and *Staphylococcus aureus *S19 and L5 showing strong support for grouping with Lactobacilliales. No inter-domain conflicts were detected*.

*It is important to note that this methodology was not designed to detect horizontal transfers; rather, simple well-supported conflicts that would violate the assumptions necessary for a concatenated ribosomal dataset*.

*As many ribosomal protein sequences are very short, there is limited phylogenetic information per protein, and the resulting tree topologies reflect this in their lack of resolution. Therefore, a stringent criterion is required for the identification of clear conflicts, as poorly supported conflicts within these trees reflect a very weak power of detection for biological events. The manuscript has been changed to communicate more clearly communicate the objectives of the conflict detection, and to elaborate on the details of the methodology. As is also now stated in the manuscript, it is important to note that the RNoL methodology is initially agnostic about "transfers" since the backbone reference tree is simply meant to be a cohesive scaffold; gene phylogenies are reconciled to this scaffold, resulting in reticulations. Only once a robust, rooted network of life is generated can something approximating a "vertical" signal be discerned (if even then), and then reticulations with respect to this history be described as horizontal gene transfers. However, this being said, it is not surprising that a technique dedicated to detecting possible transfer events (instead of highly-supported conflicts among greater taxonomic categories), would find more conflicts*.

*As far as the comment referring to evidence within *E. coli *strains for multiple histories, while transfers between closely related groups may be universally occurring at high rates, mediated by homologous recombination machinery acting on high sequence similarity, these kinds of events are omitted by the resolution of our approach, as they are not "interesting" from the perspective of deep evolutionary questions, and may fundamentally differ in mechanism*.

**Reviewer 2 continued: **The sentence "it is clear that [. . . ] a reference tree representing a history of predominantly vertical descent is an essential scaffold for any such holistic effort" is certainly correct, but maybe not as dramatically as evolutionists have long thought. First, such a unique reference tree cannot be produced for all evolving forms. Viruses and plasmids from isolated genetic worlds (see [99]) can never branch in a single vertical tree. More than one vertical tree would be required to describe their history. If the number of viruses without direct connection to the cellular gene pool increases, this genetic disconnection will increasingly become a problem. Second, the "organizing importance" of the historical tree also largely depends on the (relative) lack of information regarding other possible organizing metadata: had we more knowledge on DNA vehicles and organismal lifestyles for instance, we might decide that lifestyle is an essential scaffold for an holistic effort. Maybe it would be worth encouraging, along with the reconstruction of a rNOL, the development of additional organizing scaffolds for microbial evolution rather than to give this major role only to the history of vertical descent. Yes, history matters (we would not be evolutionists otherwise), but to what extent it is of "organizing importance" is largely an empirical question : what proportion of the genetic characters are well explained based on the vertical tree *vs *what proportion are well explained (although in different terms) using another interpretative framework [88]? In lineages with open pangenomes, lifestyle may matter more than vertical descent, at least at some scale of the analysis. Open lineages [104] will also be a problem.

What the "biological meaning" is of the central (vertical) trend is a really good question, and should be treated first like that: as a question, even though it may be tempting to assume that the vertical trend has good explanatory power. Many evolutionists hope it does, but we do not really know that. In the reconstruction of the rNOL, it should be carefully tested to what extent the gene histories are (largely) disconnected from the vertical history. In other words, maybe the authors could add some thoughts to the following issue: *Should the methodological approach to the rNOL be quite the same than the methodological approach to the TOL, or would not be additional and better congruence tests required to justify the vertical backbone? *Can the goal of obtaining a rNOL be a sufficient justification for combining sequences for improved resolution (a classical approach well described in the authors' text) without testing the congruence of these sequences? Should the assumption that there is a real meaningful vertical history recorded in the genes used to build the background be tested? It seems that rNOL builders should not rely on *a priori *assumptions about the rate of HGT of genes, and that some tests are critical. The authors have convincingly argued that, depending on the expected rate of HGT, supermatrices or supertrees should be preferred: what to do when we do not know the amount of HGT in our taxa, over time ? The transition from TOL to rNOL is largely determined by the fact that HGT may be major in some genomes and lineages, not the TOL. Thus, maybe a little section entitled 'Practical consequences of the TOL to rNOL transition" could discuss this aspect in a few sentences? If one wants to put his/her hopes in algorithmic development to improve tree reconstruction models, improved models should account for lineages with different rates of HGT (as the developments discussed in "Accounting for heterogeneous evolutionary processes" clearly indicate).

**Authors' response: ***Many interesting points are raised here. With reference to the "organizing importance" of evolutionary events, the ToL has been used to apply a strictly hierarchical classification system to extant organisms. Although we are promoting the RNoL an improved alternative phylogeny, we are not promoting a specific means of classification based on it. We agree that any felling of a ToL concept and its associated tree-thinking casts doubt over the utility of a hierarchical classification system also "rooted" in the same concept*.

**Reviewer 2 continued: **"118 species": what species? Please be precise: prokaryotes, eukaryotes?

**Authors' response: ***We sampled across available genomes of Bacteria, Archaea, and Eukaryotes to the Order and Phylum level, respectively*.

**Reviewer 2 continued: **The authors suggest that rooting the ribosomal tree of life should help by polarizing the complex reticulations of the many gene trees mapped onto it. This seems optimistic: individual gene phylogenies can be so messy (due to duplication, losses, and recombinational lateral gene transfer in addition to speciation) that even knowing how to root the ribosomal tree may not be that decisive for the polarization of these gene trees. What can be done when there are multiple copies of the same species? And why should we root patchy gene trees, for instance trees with three bacteria and one archaeon, between archaea and bacteria? Such small trees are typical outcomes of lateral gene transfers: rooting them according to the ribosomal tree of life would hide these transfers by making us believe that patchy gene families are ancestral gene families lost everywhere but in these particular lineages.

**Authors' response: ***We agree that mapping a gene tree onto the ribosomal scaffold is a complex, non-trivial process that needs to consider probabilities of gene duplications, gene loss, and gene transfer. Certainly, mapping a gene with sporadic disjoint distribution will need to incorporate gene transfer relative to the ribosomal scaffold. Furthermore, the comment on messiness is entirely correct. In many instances multiple mappings are possible, especially if extinct and unsampled lineages are taken into consideration. Especially for small gene families the distinction between gene-transfer donor and recipient often is not possible. The identification of donors and recipients is certainly probabilistic and not absolute. However, these limitations not withstanding, the availability of a rooted reference tree greatly facilitates the integration between gene and reference tree *[84,85].

**Reviewer 2 continued: **"The majority of molecular phylogenies rooted using ancient gene duplications . . .": Please remind the readers how many phylogenies did that amount to?

**Authors' response: ***The better resolved phylogenies with ancient gene duplications include the ATPase catalytic and noncatalytic subunits, several aminoacyl-tRNA synthetases, elongation factor proteins, dehydrogenases, carbamoylphosphate synthetases, and the signal recognition particle/ftsZ proteins. For details see *[26].

**Reviewer 2 continued: **There are many more examples of bacterial HGT to eukaryotes (in algae, rotifers, cnidarian), . . .

**Authors' response: ***More examples have been added to the manuscript*

**Reviewer 2 continued: **"more complex than a single tree-like narrative": I agree entirely, and you could have quoted [58] on about that topic (and other things)

**Authors' response: ***We broadly subscribe to process and "pattern pluralism", specifically that different representations of relationships will be appropriate for different purposes. We hope we have been more precise in communicating that the rooted Net of Life is intended as a phylogeny retaining the power of retrodiction where the resolution of reconstructed component gene trees allows. Other (and we would say, less narrative) ways of depicting relationships between extant organisms are certainly valuable as discussed in our response above. These approaches, such as an unrooted network with weighted edges defined by the proportion of homologous sequences shared between pairs of nodes representing genomes (*Figure 1*in *[105]*), and different approaches to extract and compare phylogenetic information retained in a set of genome *[87,88,105-108]*certainly depict evolutionary information, but largely serve a different purpose. In addition to the ribosome, other characteristics have been used to place organisms into a taxonomic framework, and, perhaps surprisingly given what we have learned about gene transfer, many of these approaches have resulted in similar groups as the ribosomal rRNA *[109]. *There is value in exploring different taxonomic classification schemes *[110]*, but here we restrict ourselves to discussing a particular phylogenetic framework, that at least initially will not impact current microbial taxonomic practice. Given that the rooted Net of Life includes reticulations, it is not intended as an explanandum for Darwin's explanans *[58].

**Reviewer 2 continued: **"if too many conflicts are present in the datasets or the phylogenetic signal is too weak [. . . ] these artifacts". Please add a few references after this sentence - there are many

**Authors' response: ***More references have been added to the manuscript*

**Reviewer 2 continued: **I understand and appreciate why the authors prefer to use the ribosomal genes over an average tree to build the vertical backbone, yet as a pluralistic thinker I would be happier if several rNOLs were reconstructed based on different vertical backbones (*i.e*. for different gene selections), so users could estimate how important the choice of the vertical backbone may be (or finally may not be) for future evolutionary conclusions.

**Authors' response: ***There is no other dataset that has as strong a signal and as biologically valid justification as the ribosome. Other backbones would likely represent more horizontal transfers between divergent organisms than the ribosomal backbone. However, there are a few systems, such as the multi-subunit V/A/F-ATPases *[111]*that have good phylogenetic resolution over most of the evolutionary history of cellular organisms. One of the first steps in implementing the RNoL concept will be to reconcile the history of these co-evolving systems of well-resolved protein-coding genes with the ribosomal scaffold*.

### Reviewer 3: Robert Beiko, Dalhousie University

In this paper, the authors describe a representation of evolution they feel would be appropriate to capture both the vertical and important lateral phylogenetic signals of gene trees. The model would use a tree based on a concatenated ribosomal dataset as a "scaffold" over which could be laid frequently observed conflicting signals à la Thermotogae, Aquificae, Thermoplasmatales, *etc*.

The idea is certainly an attractive one, but the paper is quite short on detail and I'm not sure how this model will hold up in the face of the data. Specifically:

Ribosomal proteins clearly do tend to stick together in interaction and evolutionary terms, but the statements about there being no LGT outside the order level in a whole bunch of ribo-proteins very much conflicts with our results and those of other groups. For example, the Aquificae have some ribosomal proteins that are shared exclusively with Archaea, or have strongest affinities with them. Please elaborate on your unpublished results. Are they based on a somehow restricted subset of ribosomal proteins? Did you use special reconstruction techniques (*e.g.*, correcting for compo or rate biases as alluded to later in the manuscript)? Is the result based on concatenations, or comparisons of individual gene trees?

**Authors' response: ***See response to Reviewer 2. In this way, the concatenated ribosomal tree is only special in its robust consistent phylogenetic signal, which increases confidence in reconciliation topologies. While the resulting inferences about vertical inheritance may very well map to this ribosomal tree in many instances, this is not an a priori assumption in our method, nor is it an assured outcome*.

**Reviewer 3 continued: **There is a LOT of LGT, and considering *all *lateral relationships leads to the "hazes" of the Dagan/Martin papers. Of course these trees are presented in a way to maximize the visual impact of LGT, but there is still the question of how an insane number of alternative relationships are going to be displayed on a reference backbone. Do you envision some kind of filtering procedure by which infrequent avenues of gene sharing are suppressed? Would filtering be based on numbers of events relative to genome size? Would short-distance paths (*e.g.*, within genera or named species) be suppressed since they are expected to occur for various mechanistic reasons ?

How would the tree/network actually be inferred and displayed? It is not a trivial matter to overlay a large set of reticulations onto a tree. Galled networks and cluster networks aim to do this, but even they have considerable difficulty in capturing the complex relationships among a relatively small set of trees [112].

**Authors' response: ***These are excellent points*.

*Firstly, as we have now articulated better in the manuscript, phylogenetically biased transfers occurring over "short" distances are averaged over so that sub-order relationships with potentially high frequencies of genetic exchange are not explicitly depicted*.

*On a broader scale, there may still be a sufficiently high frequency of reticulations to demand special consideration when plotting. Effectively depicting a reticulated phylogeny covering all three domains in a static two dimensional figure probably is not possible. A filtering procedure is a good idea, perhaps in the context of a computer based interactive graphical display so that levels of detail can be adjusted for clarity when viewing a particular part of the model. A range of filtering criteria could be implemented including, where known, inferred function, distance over vertical edges, frequency between certain lineages. Using a range of filtering criteria could also be adapted to inferring the nature of biases (including more frequent avenues) among certain gene families and between certain lineages*.

**Reviewer 3 continued: **"...the ToCD is only knowable insofar that a vertical signal is preserved..." To this I would add "and identifiable as such". It very well may be that whatever extant set of organisms are the closest cellular sisters to the Aquificae do indeed share some phylogenetic affinities with them, but short of privileging certain molecular systems such as the ribosome or cell wall synthesis, it is statistically very difficult to decide which of the phylogenetic affinities, none of which constitutes a majority of the overall signal, is the one to be pinned down as "sister" to the Aquificae.

**Authors' response: ***We agree that it has not yet been proven beyond reasonable doubt that the Aquificales are not epsilonproteobacteria that picked up a ribosome from an ancient lineage by HGT. The assumption that the ribosome of the Aquficales and Thermotogales reflects their vertical ancestry indeed reflects bias in considering the phylogenetic import of particular molecular systems. We note that this bias is not a prerequisite for reconstructing the RNoL; however, it does influence the interpretation. There is no *a priori *reason why such bias is unreasonable or undesirable, provided it is not arbitrary; even in traditional taxonomies, the usefulness of characters is evaluated based on their utility in defining groups, frequency of gain/loss, or ease of identification. In the light of gene-based phylogenies and horizontal transfer, the problem therefore appears to be that no quantitative, objective means yet exists for weighing the often disparate phylogenetic signals inferred for different parts of the molecular machinery. It is clear that different kinds of genes are transferred with different frequencies between groups at varying taxonomic levels, and that this is influenced by protein function, the structure of macromolecular systems, as well as other factors. While beyond the methodologies and scope of this manuscript, once a rNOL is constructed, a carefully developed set of such criteria could be used to evaluate reticulations, determining to what degree signals reflect vertical descent, artifacts, noise, highways of gene transfer, or other patterns of inheritance. For now, while the choice of the ribosome is arbitrary in the absence of initial assumptions of vertical *vs*. horizontal inheritance, it is deliberate in the cohesive, robust signal it represents, which is necessary in a scaffold*.

**Reviewer 3 continued: **"The transfer of an entire ribosome..." Wait, doesn't this invalidate the whole model and contradict what you have been saying for the entire manuscript? Many of the concatenated ribo analyses (*e.g.*, Boussau et al. 2008, which you cite) ultimately make some assertion that the ribosome is king, and that *this *signal is the one that must be correct, even in the face of overwhelming evidence from other gene trees and systems. To continue beating the unicellular, hyperthermophilic Aquifex horse, most molecular systems (*e.g.*, broken out by COG category) favour Epsilonproteobacteria-Aquificae linkages rather than the canonical, ribosomal Aquificae+Thermotogae story. What would it take, then, to convince someone that the ribosome really *has *been transferred, and that Aquificae+Epsilonproteobacteria is "real"?

**Authors' response: ***In the original abstract where we said "predominantly vertical lines of descent" and in the introduction where we said "the mostly vertical evolutionary descent of a coherent biological entity" with respect to the ribosome phylogeny scaffold, we were anticipating that a ribosome would prove to be rarely transferred for the reasons discussed below. We realize this speculation may have been unhelpful and have made revisions emphasizing that vertical inheritance of the ribosome need not be the rule. We also realize the sub-heading "The Reticulated Ribosomal Tree" was positively misleading (reticulations are only labeled HGTs given sufficient evidence) and apologize accordingly! Our speculation that total ribosomal transfer is extremely unlikely, was due to these reasons:*

1. Several operons (of both protein and RNA) would all have to be transferred, involving many many kilobases of sequence and numerous independent events;

2. Ribosomal components are highly expressed, and for all these dozens of extra proteins and large RNAs, the cellular economy would provide strong selection against their successful transfer unless there was some major advantage;

3. What major advantage could an entire transfer provide? Antibiotic resistance could be achieved by the transfer of single riboproteins, in most cases;

4. Having two functional ribosomes with so many highly similar, but slightly different subunits floating around would likely poison both assembly processes and be extremely lethal;

5. Since the native ribosome must be lost, and this can't happen without the new one being replaced, both must be expressed at the same time, but see (4);

*6. In the case that subunits are compatible enough to avoid toxicity, then one would expect more random subunit loss resulting in a hybrid ribosome. This is not observed*.

*Data that would convince us of a ribosomal transfer to the ancestor of the Thermotogales or Aquificales would be a strong coherent signal for many other genes placing a large part of the remainder of the genome at a single point*, e.g., *a finding that the majority of genes in the Thermotogales appear specifically related to the Thermoanaerobacter lineage would support these as a possible sistergroup to the Thermotogales in a tree of cell division. However, this is not what we observe. If the ribosome were transferred in a trickle down fashion (see above) then different signals for different ribosomal components might be detected. Our preliminary data suggest the opposite, that genes from clostridia and archaea appear to be continuously acquired in the different lineages of the Thermotogales. In contrast, the ribosomal components contain a weak but consistent signal that is reinforced as more ribosomal components are added to the analysis*.

**Reviewer 3 continued: **A self-serving comment: our 2008 paper in Systematic Biology [61] dealt extensively with the averaging of phylogenetic signals that goes on in genome phylogeny analysis; it may be worth citing in the discussion of phylogenetic signal averaging, since it demonstrates that the robustness of inference is highly dependent on both the rate and regime of LGT.

**Authors' response: ***We added this citation to the discussion*

**Reviewer 3 continued: **Finally, a grammatical comment: Compound adjectives must be hyphenated, *e.g. *"genome-wide analyses" and elsewhere.

Italicize "Methanosarcina mazei".

**Authors' response: ***We changed the text as suggested*.
